# UNMET NEEDS IN SELF-DIRECTED HCBS PROGRAMS

**DOI:** 10.1093/geroni/igz038.859

**Published:** 2019-11-08

**Authors:** Kevin J Mahoney, Ellen k Mahoney, Carmen Morano, Andrew DeVellis

**Affiliations:** 1 Boston College School of Social Work National Resource Center (NRCPDS), Chestnut Hill, Massachusetts, United States; 2 Connell School of Nursing, Boston College, Chestnut Hill, Massachusetts, United States; 3 School of Social Welfare, University at Albany, State University of New York, Albany, New York, United States; 4 Simmons School of Social Work, Boston, Massachusetts, United States

## Abstract

Unmet Need for long-term services and supports has been linked to a variety of harmful health outcomes. One suggested strategy for ameliorating unmet need is to give participants control of the budget and let them construct individualized plans. The evaluation of the Cash and Counseling controlled experiment documented a marked reduction in unmet need when compared to traditional agency-based solutions, but it also showed significant unmet needs remained. This paper, drawing from 76 ethnographic case studies of Cash and Counseling participants, gives us an understanding of what those unmet needs are, who sees them, and what participants and their family caregivers see as possible remedies. Certain areas of unmet need identified in this study stand out. These included health-related problems, environmental issues, and the caregivers’ need for relied. The paper concludes with implications for care integration and the training of support brokers as warnings about reducing budgets.

